# Ocular injuries among patients with major trauma in England and Wales from 2004 to 2021

**DOI:** 10.1038/s41433-024-03116-y

**Published:** 2024-05-24

**Authors:** Mohammed Talha Bashir, Omar Bouamra, James F. Kirwan, Fiona E. Lecky, Rupert R. A. Bourne

**Affiliations:** 1 School of Clinical Medicine, University of Cambridge, Cambridge, UK; 2 The Trauma Audit & Research Network, University of Manchester, Manchester, UK; 3 Department of Ophthalmology, Queen Alexandra Hospital, Portsmouth, UK; 4 Centre for Urgent and Emergency Care Research (CURE), School of Health and Related Research, The University of Sheffield, Sheffield, UK; 5 Department of Ophthalmology, Cambridge University Hospitals, Cambridge, UK; 6 Vision & Eye Research Institute, School of Medicine, Anglia Ruskin University, Cambridge, UK

**Keywords:** Trauma, Epidemiology, Epidemiology

## Abstract

**Background:**

Ocular trauma is a significant cause of blindness and is often missed in polytrauma. No contemporary studies report eye injuries in the setting of severe trauma in the UK. We investigated ocular injury epidemiology and trends among patients suffering major trauma in England and Wales from 2004 to 2021.

**Methods:**

We conducted a retrospective study utilising the Trauma Audit and Research Network (TARN) registry. Major trauma cases with concomitant eye injuries were included. Major trauma was defined as Injury Severity Score >15. Ocular injuries included globe, cranial nerve II, III, IV, and VI, and tear duct injuries. Orbital fractures and adnexal and lid injuries were not included. Demographics, injury profiles, and outcomes were extracted. We report descriptive statistics and 3-yearly trends.

**Results:**

Of 287 267 major trauma cases, 2368 (0.82%) had ocular injuries: prevalence decreased from 1.87% to 0.66% over the 2004–2021 period (*P* < 0.0001). Males comprised 72.2% of ocular injury cases, median age was 34.5 years. The proportion of ocular injuries from road traffic collisions fell from 43.1% to 25.3% while fall-related injuries increased and predominated (37.6% in 2019/21). Concomitant head injury occurred in 86.6%. The most common site of ocular injury was the conjunctiva (29.3%). Compared to previous TARN data (1989–2004), retinal injuries were threefold more prevalent (5.9% vs 18.5%), while corneal injuries were less (31.0% vs 6.6%).

**Conclusions:**

Whilst identifying eye injuries in major trauma is challenging, it appears ocular injury epidemiology in this setting has shifted, though overall prevalence is low. These findings may inform prevention strategies, guideline development and resource allocation.

## Introduction

The Global Burden of Disease Study estimated that almost 60 million cases of ocular trauma occurred worldwide in 2019 and these were associated with over 400,000 years lived with disability [1]. Ocular trauma is a major cause of visual impairment, and 1.6 million cases of blindness can be attributed to eye injuries globally [2]. These injuries also carry a significant economic burden. In the USA, the inpatient cost of ocular trauma admissions from 2001 to 2014 was estimated to be 1.72 billion dollars [3], and further costs are associated with productivity losses [4].

Major trauma is the leading cause of morbidity and mortality in those under the age of 40 [5]. Ocular trauma is common among these patients and is associated with vision-threatening pathology [6–11]. However, these injuries are often missed as primary surveys prioritise life-threatening pathology, and examination of the visual system in these patients, who are often unconscious or have concurrent periorbital injuries, is challenging [11–13].

Major trauma was historically thought to primarily affect young men involved in road traffic accidents, however, significant demographic shifts have occurred in the UK in recent decades. Older adults are emerging as the major part of the population and low-energy falls are now more prevalent [14]. Improvements in outcomes have also been seen in these patients following the remodelling of major trauma care in England and Wales [15].

There are limited data on ocular injuries in the context of major trauma [6–10, 16–22], and none of these studies have investigated changes over time within a defined population. The most recent UK-based study examining ocular trauma in this setting was almost 20 years ago [8], and little is known about current demographics and injury patterns. To address this, we explored a major UK trauma database to analyse epidemiology and trends in ocular injuries in patients with major trauma from 2004 to 2021. We also compared data on the anatomical location of ocular injuries with the data from a previous survey (1989–2004), which used identical methodology [8].

## Methods

A registry-based study was conducted using data held by the Trauma Audit Research Network (TARN). TARN is a national trauma registry that aggregates and reports data for all trauma-receiving hospitals in England and Wales. It includes patients who are admitted to hospital for ≥72 h, require critical care support, are transferred to a tertiary/specialist centre, or who die from their injuries and excludes patients aged over 65 with an isolated fracture of the femoral neck or pubic ramus and those with single uncomplicated limb injuries. TARN records data up to 30 days of hospital admission.

After a patient is identified for inclusion into TARN, a dataset of prospectively recorded variables is collated by local coordinators using a standard web-based case record form. Injury descriptions are generated from multiple sources including clinician’s notes, discharge summaries, and imaging, operative, and necropsy reports. All injuries are then coded centrally using the Abbreviated Injury Scale (AIS) which is an anatomical-based coding system that also grades the severity of each injury on a six-point scale, AIS 1 describes minor injuries and AIS 6 describes injuries which are incompatible with life [23]. The Injury Severity Score (ISS) is then calculated by adding the squares of the highest scoring injury from the three most injured body regions. ISS is used to grade the overall trauma severity and cases with an ISS of 16 or more are considered major trauma.

We included all cases of major trauma from 2004 to 2021 with any concomitant AIS codes relating to injuries to the globe, tear duct, or to the second, third, fourth, or sixth cranial nerves. Orbital fractures and adnexal injuries (other than tear duct injuries) were not counted as ocular injuries and were instead coded as facial injuries. Eyelid injuries were also not included as these are not coded by the AIS. Extracted data for each patient included demographic information, a complete injury profile, hospital course, and mortality.

Data, other than the anatomical location of ocular injury, were summarised for each 3-year window, e.g. 2004–2006 as percentages and medians, and trends were analysed using the Chi-square test for trend and the Jonckheere–Terpstra test, for categorical data and non-normally distributed numerical data as appropriate. Missing data were not included in analyses and a two-sided *p*-value of 0.05 was considered statistically significant. Ocular injuries over the 2004–2021 period were anatomically classified and tabulated (e.g. injury to the cornea/retina) and each injury was counted separately such that a case may include more than one injury per eye. The spectrum of injuries was compared simply with the previous TARN study investigating ocular injuries in major trauma from 1989 to 2004 [8]. All analyses were carried out using R version 4.1.1 (R Core Team 2022) [24].

TARN analyses anonymised data with permissions granted by the Health Research Authority Clinical Advisory Group (CAG–PIAG Section 251). This project was registered within TARN (Project ID INC-117).

## Results

**Fig. 1:**
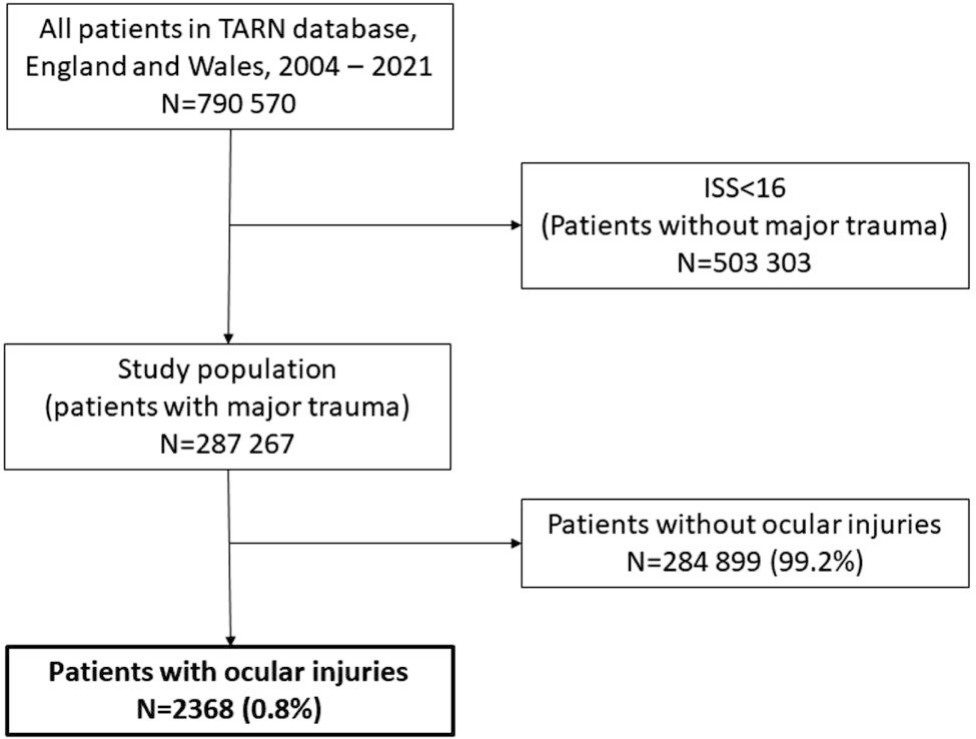
PRISMA flow diagram showing case ascertainment from TARN 2004–2021. *TARN* Trauma Audit Research Network, *ISS* Injury Severity Score.

Figure 1 displays how the study sample was ascertained from the TARN database. TARN included an increasing number of cases of major trauma between 2004 and 2021 (Table 1) totalling 287,267 cases over the 18 years (Fig. 1). 2368 (0.82%) had eye injuries and this proportion decreased over the study period (1.87% in 2004/06 and 0.66% by 2019/21) (*p* < 0.0001) (Table 1). Males comprised 72.2% of eye injury cases and the median age was 34.5 years which showed a small but statistically significant rise (τb = 0.079, *p* < 0.0001). 93.9% of injuries were due to blunt trauma. The median ISS was 26 and mortality was 15.1% which remained stable over the 18 years.

**Table 1:** TARN population of cases of major trauma with concurrent ocular injuries in England & Wales, 2004–2021.

	2004/06	2007/09	2010/12	2013/15	2016/18	2019/21	Total	*p* value
Ocular injury (*n*)/all major trauma *(n) (%)*	167/8907 (1.87%)	180/14,242 (1.26%)	365/35,321 (1.03)	465/59,495 (0.78%)	611/81,079 (0.78%)	580/88,223 (0.66%)	2368/287,267 (0.82%)	<0.0001^a^
Age, median (IQR)	34.5 (12.7–45.9)	30.2 (18.1–48)	28.7 (1.1–53.4)	32.1 (0.5–60.2)	34.9 (0.7–64.1)	40 (19.8–66.3)	34.5 (3–59.5)	<0.0001^b^
Male, *n* (%)	136 (81.4%)	138 (76.7%)	270 (74%)	345 (74.2%)	412 (67.4%)	409 (70.5%)	1710 (72.2%)	0.003^a^
GCS, median (IQR)	14 (7–15)	14 (9–15)	14 (8–15)	14 (8–15)	14 (9–15)	14 (8–15)	14 (8–15)	0.075^b^
ISS, median (IQR)	26 (19–29)	26 (19–34)	26 (20–30)	27 (21– 33)	27 (21–33)	27 (21–33)	26 (21–33)	<0.0001^b^
Mechanism of injury: *n* (%)								
RTC	72 (43.1%)	85 (47.2%)	122 (33.4%)	121 (26%)	156 (25.5%)	147 (25.3%)	703 (29.7%)	<0.0001^a^
Fall > 2 m	15 (9%)	21 (11.7%)	37 (10.1%)	45 (9.7%)	51 (8.3%)	72 (12.4%)	241 (10.2%)	
Fall < 2 m	12 (7.2%)	10 (5.6%)	45 (12.3%)	75 (16.1%)	115 (18.8%)	146 (25.2%)	403 (17%)	
Assault	13 (7.8%)	20 (11.1%)	59 (16.2%)	50 (10.8%)	73 (11.9%)	65 (11.2%)	280 (11.8%)	
NAI < 16 yrs	25 (15%)	25 (13.9%)	78 (21.4%)	126 (27.1%)	162 (26.5%)	94 (16.2%)	510 (21.5%)	
Other	30 (18%)	19 (10.6%)	24 (6.6%)	48 (10.3%)	54 (8.8%)	56 (9.7%)	231 (9.8%)	
Injury type: *n* (%)								
Blunt	153 (91.6%)	168 (93.3%)	338 (92.6%)	441 (94.8%)	575 (94.1%)	548 (94.5%)	2223 (93.9%)	0.581^a^
Penetrating	14 (8.4%)	12 (6.7%)	27 (7.4%)	24 (5.2%)	36 (5.9%)	32 (5.5%)	145 (6.1%)	
Concomitant serious (AIS 3+) injuries: *n* (%)								
Head	138 (82.6%)	149 (82.8%)	322 (88.2%)	410 (88.2%)	525 (85.9%)	506 (87.2%)	2050 (86.6%)	0.238^a^
Face	30 (18%)	41 (22.8%)	29 (7.9%)	23 (4.9%)	32 (5.2%)	42 (7.2%)	197 (8.3%)	<0.0001^a^
Chest	44 (26.3%)	54 (30%)	112 (30.7%)	139 (29.9%)	156 (25.5%)	171 (29.5%)	676 (28.5%)	0.443^a^
Abdomen	11 (6.6%)	9 (5%)	19 (5.2%)	19 (4.1%)	31 (5.1%)	22 (3.8%)	111 (4.7%)	0.665^a^
Spine	6 (3.6%)	13 (7.2%)	15 (4.1%)	45 (9.7%)	94 (15.4%)	58 (10%)	231 (9.8%)	<0.0001^a^
Pelvis	11 (6.6%)	19 (10.6%)	17 (4.7%)	15 (3.2%)	25 (4.1%)	21 (3.6%)	108 (4.6%)	0.001^a^
Limb	18 (10.8%)	25 (13.9%)	29 (7.9%)	53 (11.4%)	60 (9.8%)	43 (7.4%)	228 (9.6%)	0.071^a^
Interventions								
Any operation, *n* (%)	59 (35.3%)	82 (45.6%)	132 (36.2%)	161 (34.6%)	202 (33.1%)	218 (37.6%)	854 (36.1%)	0.063^a^
Time to any operation (hours), median (IQR)	9 (2.7–30.6)	18.6 (4.7–89)	8.6 (3.8–31.4)	16.3 (3.4–71.1)	14.5 (3.1–59.6)	13.1 (4.4–37)	13.2 (3.6–47)	0.744^b^
Eye operation, *n* (%)	2 (1.2%)	1 (0.6%)	6 (1.6%)	21 (4.5%)	43 (7%)	29 (5%)	102 (4.3%)	<0.0001^a^
Time to eye operation (hours), median (IQR)	58.8 (58.8–58.8)	145 (145–145)	3.6 (2.7–8.6)	18.2 (11.7–51.9)	13.3 (4.7–23)	17.2 (7.1–34.5)	15.4 (5.5–32.7)	0.845^b^
Outcome								
Mortality, *n* (%)	18 (13.2%)	13 (8.8%)	37 (14.1%)	58 (17.5%)	67 (14.9%)	74 (16.6%)	267 (15.1%)	0.193^a^
LOS (days), median (IQR)	11 (5–25)	12 (5.5–24)	11 (4–21)	10 (4–20)	9 (4–22)	9 (4–19)	10 (4–21)	0.002^b^

*TARN* Trauma Audit Research Network, *IQR* interquartile range, *GCS* Glasgow Coma Scale, *ISS* Injury Severity Score, *RTC* road traffic collision, *NAI* nonaccidental injury, *AIS* Abbreviated Injury Score, *LOS* length of stay.

^a^Chi square for trend.

^b^Jonckheere–Terpstra test.

Road traffic collisions (RTCs) were the most common mechanism of major trauma involving ocular injury in 2004/06 accounting for 43.1% cases but decreased to 26% by 2013/15. Conversely, falls became more prevalent in the dataset and, from 2016/18, were the most common mechanism of injury (27.1%). Low-energy (<2 m) falls comprised the majority of these. The prevalence of nonaccidental injuries (NAIs) in children under 16 also increased from 15% in 2004/06 and accounted for as much as 27.1% of cases in 2013/15, however, this fell to 16.2% during the 2019/21 period.

Head and chest trauma comprised the most common serious (AIS 3+) concomitant injuries. These were present in 86.6% and 28.5% of cases respectively and remained stable over the period. Serious facial injuries were present in 18% of cases in 2004/06 and prevalence fell to 7.9% in 2010/12 from when it remained stable.

4.3% of patients required an eye operation and this increased over time (*P* < 0.0001) while the proportion of patients requiring *any* surgical intervention was 36.1% and remained stable. Median time to ophthalmic surgery was 15.4 hours which was similar to the 13.2 hours for *any* operation.

The most common injuries over the 2004–2021 period were to the conjunctiva (29.3%), retina (18.5%), and sclera (12.8%) (Table 2). 8.6% of injuries were of the optic nerve and injuries to cranial nerves III, IV, and VI comprised 8.6%, 0.9%, and 5.5%, respectively. There were more injuries recorded in TARN in the 2004–2021 period compared with the 1989–2004 study [8]. Comparing spectrum injuries, notably corneal injuries were less prevalent in our contemporary study, and retinal and conjunctival injuries were more prevalent.

**Table 2:** Spectrum of ocular injuries sustained. TARN 1989–2004 compared with TARN 2004–2021.

Ocular injury^a^	Ocular injuries: TARN 1989–2004 *n* (%)^b^	Ocular injuries: TARN 2004–2021 *n* (%)
Tear duct	3 (0.3%)	13 (0.4%)
Conjunctiva	127 (12.9%)	941 (29.3%)
Cornea	305 (31.0%)	211 (6.6%)
Uvea/Iris	7 (0.7%)	16 (0.5%)
Vitreous	15 (1.5%)	107 (3.3%)
Retina	58 (5.9%)	595 (18.5%)
Choroid	3 (0.3%)	15 (0.5%)
Sclera	90 (9.1%)	412 (12.8%)
Eye avulsion	40 (4.1%)	30 (0.9%)
Eye (not further specified)	80 (8.1%)	113 (3.5%)
Optic nerve	130 (13.2%)	276 (8.6%)
Oculomotor nerve	71 (7.2%)	276 (8.6%)
Trochlear nerve	12 (1.2%)	28 (0.9%)
Abducens nerve	43 (4.4%)	176 (5.5%)
Total	984 (100%)	3209 (100%)

^a^Patients can have more than one eye injury and more than one injury per eye.

^b^From Guly et al. [8].

Data were complete for all variables except Glasgow Coma Scale measurements which were recorded in 2074 (87.6%) cases.

## Discussion

We present contemporary data, from a large national trauma registry, for ocular injuries in the setting of major trauma in England and Wales. Ocular injuries occurred in 0.8% of patients with major trauma which is lower than other studies which report rates between 2.3% and 16% [6–11]. This can be partly due to lid and adnexal injuries, and orbital fractures not being classified as ocular injuries in our cohort; whereas they were included in other studies. The prevalence of ocular injuries in this cohort decreased over the 18-year period and was less than the 2.3% reported in the 1989 to 2004 study of TARN data using identical case ascertainment criteria [8]. This apparent decline in eye injuries is likely multifactorial and may be partially explained by nationwide shifts in injury mechanism—there has been a significant reduction in RTC-related major trauma, which historically resulted in the most ocular injuries [8, 14]. Additionally, this may represent a relative decrease in proportion of ocular injuries as the identification of major trauma, particularly among older adults, has improved. These improvements have been attributed to the advent of major trauma networks in 2012 and to the now widespread use of whole-body CT scanning in polytrauma which allows for the detection of injuries that previously may have remained occult [14, 25]. Hospital Episode Statistics data, which is more comprehensive, has been interrogated in other studies which report a large absolute increase in cases coded as major trauma in patients in England during the study period [14, 15].

The demographics of major trauma in the UK have changed significantly in recent decades. RTC-related injuries have halved, and an ageing population has been associated with an increase in injuries secondary to low falls which are emerging as the predominant mechanism [14]. In our study, RTCs accounted for as much as 47.2% of cases in 2007/09 and almost halved to 25.3% in 2019/21. These trends are likely owing to improvements in vehicle and road safety [14] and similar findings have been demonstrated in ocular injury incidence across Western Europe and in other high-income regions [1, 26]. A reduction in RTC-related injuries may also account for the decline in patients with concomitant facial and pelvic injuries in our cohort as these are commonly associated with this injury mechanism [27]. Ocular injuries secondary to low falls in this setting increased significantly over the 18-year period from 7.2% to 25.2% and similar trends have been reported in other studies [1, 28–31]. These injuries are common among older adults however we did not see a clinically meaningful increase in age over the same period, contrary to what has been reported in other literature [28, 31]. It may be that any rise in older adults sustaining these injuries in our data was offset by the increase in children suffering NAI. Falls in older adults are associated with ocular contusions, eyelid lacerations, and open globe injuries [29] and better understanding of falls-related injury will enable improved recognition and management of ocular trauma in a patient group which is projected to continue to increase [32].

Head injury is prevalent in major trauma [33] and was the most common serious (AIS 3+) concomitant injury in this study, present in 86.6% of cases of ocular trauma. The relationship between eye injury and head trauma is well described with ocular injuries present in 2.2–25.3% of these patients [34, 35]. Ocular injuries in this setting are frequently associated with RTCs and most commonly comprise anterior segment and neuro-ophthalmic injuries [35]. Identification of eye injuries in this cohort, however, is challenging, notably in the acute setting when patients may be agitated or unconscious. Ocular assessment in primary surveys, which prioritise recognising life-threatening injuries, is limited to assessing eye-opening and pupillary response and identifying nerve palsies, which are indicators of brain injury severity and are predictive of mortality [36].

A significant and increasing proportion of cases were children who had suffered injuries secondary to nonaccidental trauma. TARN does not report any concurrent rise in suspected physical abuse or assault in children with major trauma over the study period [37]. The reasons for this trend are unclear but may be due to increased recognition and reporting of NAI in children with eye injuries as up-to-date guidance is developed [38, 39] and reporting mechanisms are established [40]. Ocular pathologies are common in child abuse and may be varied in their presentation [41]. Retinal haemorrhages have been shown to have a high sensitivity and specificity for abusive head trauma in infants and, when accompanied with intracranial injuries, are pathognomonic [42]. ICON, a campaign aimed at preventing abusive head trauma in infants was launched in 2020 [43] and may have contributed to the reduction in NAI-related eye injuries seen in the 2019/21 period.

The most common injuries were sustained to the conjunctiva (29.3%) and likely comprised minor injuries like subconjunctival haemorrhages and conjunctival lacerations. When compared with the previous (1989–2004) TARN study, corneal injuries have fallen dramatically from 31% to 6.6% and retinal injuries have increased threefold from 5.9% to 18.5%. One contributing factor may be developments in vehicle safety. Airbags are commonly associated with corneal injuries which frequently occurred in RTCs when the occupant would meet the airbag during the inflation phase [44–46]. Older designs deployed at very high speeds and had long inflation times, subsequent versions were developed to use less power and inflate more evenly [44]. Modern airbags now employ smart technology to moderate the strength of deployment depending on the weight and position of the occupant and have been suggested to contribute to a fall in RTC-related eye injuries in the USA [26]. Furthermore, it has been proposed that seamless airbags, which are now common in modern cars, may mitigate corneal injuries often caused by abrasive fabrics found on seamed designs [44]. Improvements in vehicle safety and road design have also resulted in more patients surviving high-speed RTCs [47]. Such high-velocity trauma is more likely to result in traumatic retinopathy [48] and this may have contributed to the relative increase in retinal injuries. These injuries include traumatic retinal detachments for which urgent surgery is indicated and an increase in traumatic retinopathy may have contributed to trends in ophthalmic procedures which rose over the study period. Another contributing factor to the rise in retinal injuries may be the increase in recorded cases of NAI in children in which injuries such as retinal haemorrhages are commonly sustained [42].

While optic nerve and retinal injuries are often vision threatening, we do not report visual outcome data for cases. Among major trauma victims, those with lid lacerations, open globe injuries, and optic nerve injuries are reported to have the worst visual outcomes [6, 7]. Clinically, the Ocular Trauma Score is a tool used to predict functional outcomes after eye injury [49] and has been validated in major trauma [6], however its parameters include initial visual acuity and presence of afferent pupillary defect, which are challenging to ascertain in patients with impaired cognition, and periorbital swelling respectively [12]. In these situations, CT scanning has been shown to be useful in the identification of vision-threatening pathology such as open globe injury [50] and an intact globe on imaging is unlikely to need immediate surgical intervention [51]. CT has also been validated for the prognostication of ocular injuries and markers such as loss of globe integrity and optic canal and nerve injury are independent predictors of poor visual outcomes [52]. Machine learning and big data analytics are being utilised to construct new prognostic tools, such as the Ocular Trauma Score-2, which may have improved utility in patients with multiple injuries [53].

As eye injury epidemiology is shifting, effective prevention strategies must be developed. RTC-related major trauma, and resultant eye injuries, have fallen significantly in the UK and in many other high-income regions but continue to rise in other settings globally [1]. RTC prevention strategies adopted by the UK during this time include policy change in the form of the 2006 Road Safety Act [54] which introduced several measures including a new driving penalty system and the creation of new road offenses, and these measures were bolstered by strong law enforcement services and nationwide education campaigns. Other studies report that enforcement interventions, such as speed cameras and police patrols, are most effective at reducing the number of RTCs [55] and such prevention strategies may be adopted in settings where the burden of RTCs is higher. In the UK, eye injury prevention strategies should target falls prevention and common approaches in older adults include physical exercise and environmental modifications such as the installation of nonslip mats and grab rails in bathrooms where falls frequently occur [56]. Low vision is a risk factor for falls [57], ophthalmologists should play an active role in falls prevention by educating at-risk groups and by optimising services such that reversible causes of low vision, such as cataract, are treated in a timely manner.

The identification and management of ocular trauma in patients with multiple injuries remains a challenge in the emergency setting however excellent guidance has been published on the topic [12]. Advanced Trauma Life Support principles should be followed for all patients with major trauma [58]. Visual pathways injuries should be screened for in patients with head or facial trauma and early surveys should arguably be able to exclude a sight-threatening injury requiring emergent management, such as retrobulbar haemorrhage, even in unresponsive patients [12]. CT scanning, which is now standard in polytrauma, is sensitive and specific for many types of eye injuries and ocular and orbital CT should be undertaken in all patients with clinical suspicion [59].

## Strengths and limitations

TARN is a large, long-running registry with rigorous case identification and description processes providing high quality, representative data. However, there are several limitations that caveat our findings. Firstly, the TARN registry has grown significantly in size over the study period owing to an increase in submitting hospitals since regional major trauma networks were established around 2012 and submission to TARN became mandatory for all trauma-receiving units, better case ascertainment secondary to Best Practice Tariffs, and improvements in major trauma identification owing to more prevalent imaging [14, 15]. Changes in case ascertainment have been associated with an unmasking of “Silver Trauma” (major trauma in adults over 65) which may contribute to some of the trend data we report, although many of our findings are in keeping with other contemporary literature [1, 26, 28, 31]. Secondly, patients in whom injuries are identified after discharge, or after the 30-day TARN data collection period are not included. This may represent a significant proportion of patients as detailed ocular assessment may not be practicable in the early phase of admission in major trauma and many ophthalmic injuries are only identified in an outpatient setting after discharge. There are also limitations associated with the use of a non-ophthalmic trauma registry. Injury data are reported as anatomical location and injury type (blunt vs penetrating) and these do not carry the same clinical relevance as specialised classifications such as the Birmingham Eye Trauma Terminology System which is widely used [60]. Furthermore, important prognostic information such as visual acuity and the presence or absence of relative afferent pupillary defect are not recorded. Registries that are specialised for ophthalmic pathologies collect and code clinically relevant data and generally avoid these limitations. Examples of these include the Intelligent Research in Sight (IRIS®) Registry which was established in 2014 and has produced important data with outcomes not routinely recorded in trauma registries [61–63], and more recently, the International Globe and Adnexal Trauma Epidemiology Study Registry which has been established as a specialist registry with a focus on ocular trauma [53].

## Conclusion

In conclusion, we report information about the epidemiology of ocular injuries, in the setting of major trauma, in England and Wales, over an 18-year period. Ocular injuries are present in 0.8% of cases of major trauma and the most common concomitant injuries are to the head. RTCs are no longer the dominant mechanism of injury, which is being overtaken by falls, a pattern that mirrors major trauma trends across the UK. Fewer patients seem to be suffering corneal injuries and there has been a relative increase in retinal injuries and those undergoing ophthalmic surgery. Eye injury prevention strategies, in this setting, should target those at the highest risk of falls-related injuries, such as older adults and those with low vision. The findings of this study are important for health providers across the UK and may inform guideline development, resource allocation, and training priorities.

## Summary

### What was known before

- Eye injuries are common but are often occult in patients suffering major trauma. Major trauma demographics have changed in the UK, but no recent studies have reported on ocular injuries in this population.

### What this study adds

- Our study provides contemporary data on epidemiology and 18-year trends in ocular trauma amongst major trauma patients in England and Wales. Prevalence of ocular injury in this setting has decreased. The predominant injury mechanism in these patients has shifted from road traffic collisions to falls. These patients are suffering fewer corneal injuries, and more retinal injuries and are undergoing more ophthalmic procedures.

## Data Availability

Data are not currently available whilst TARN transitions to the National Major Trauma Registry (NHS-England) please contact Professor Fiona Lecky (f.e.lecky@sheffield.ac.uk) for updates.
